# Oxidation Behavior of Direct Reduced Iron Powder During Ball-Milling Treatment

**DOI:** 10.3390/ma19071369

**Published:** 2026-03-30

**Authors:** Qiao Liu, Zhikai Liang, Cheng Zhang, Xinyu Fu, Lingyun Yi, Zhucheng Huang, Jiayuan Li, Jun Chen

**Affiliations:** 1 Hunan Provincial Key Laboratory of Xiangnan Rare-Precious Metals Compounds and Applications, School of Chemistry and Environmental Science, Xiangnan University, Chenzhou 423000, China; xnxycz@163.com (Q.L.);; 2 School of Minerals Processing & Bioengineering, Central South University, Changsha 410083, China

**Keywords:** oxidation behavior, direct reduced iron powder, wet grinding mill, nanoscale surface, electrochemical/atmospheric corrosion

## Abstract

High-quality direct reduced iron (DRI) powder is essential for applications in catalysis, adsorption, and electromagnetic materials. However, its tendency to reoxidize during processing presents a significant challenge. This study investigates the oxidation behavior of DRI powder during wet ball-milling treatment. Samples were characterized using chemical phase dissolution, X-ray diffraction (XRD), scanning electron microscopy with energy-dispersive X-ray spectroscopy (SEM-EDS), and X-ray photoelectron spectroscopy (XPS) to assess both bulk and surface oxidation. The results reveal that significant oxidation occurs during the wet grinding and subsequent processing stages, with the relative oxidation degree (ROD) of the iron powder increasing sharply from 6.08% to 26.81% as the grinding time is extended from 20 to 40 min. SEM-EDS analysis indicates that oxidation is particularly pronounced in particles smaller than 10 μm. XRD confirms the gradual transformation of Fe^0^ to Fe_3_O_4_ with prolonged grinding, corroborating the chemical analysis. Furthermore, XPS analysis of the Fe 2p, Fe 3p, Fe 3s, and O 1s core levels reveals that the nanoscale surface is composed of Fe_2_O_3_, Fe_3_O_4_, Fe(OH)_3_, and FeOOH—a composition distinctly different from the bulk Fe/Fe_3_O_4_ phases. These findings underscore the critical roles of particle size and mechanical activation in driving DRI reoxidation during wet milling.

## 1. Introduction

The growing demand for high-quality raw materials has driven significant research into advanced beneficiation methods for low-grade, complex iron ores to meet the requirements of the steel industry [1,2]. China, despite possessing vast iron ore reserves, faces challenges due to the low grade (20–50% Fe) and high silica content of its ores, which render traditional mineral processing technologies inadequate [3,4,5].

To address these issues, we previously developed and industrially tested a novel coal-based direct reduction process using ore-coal composite mini-pellets in a low-temperature rotary kiln (≤1250 K) [6,7]. This process efficiently produces direct reduced iron (DRI) mini-pellets. However, regardless of the reduction route—whether coal-based or hydrogen-based—the resulting DRI powder is prone to rapid reoxidation during subsequent wet grinding, which adversely affects the quality of the final iron powder [8,9].

While the low-temperature interaction of oxygen with iron and steel surfaces has been extensively studied [10,11,12,13,14,15], these investigations often focus on idealized, static conditions. Recent work has also begun to address the reoxidation of H_2_-reduced iron under ambient conditions [16]. The proposed mechanisms—ranging from electric-field-driven oxidation [17] to atmospheric corrosion limited by oxygen diffusion [18,19]—typically describe slow, passive film formation. In contrast, the reoxidation rate of DRI powder during wet grinding can be dramatically accelerated. This discrepancy suggests that the mechanical activation and continuous generation of fresh surfaces in a wet, aerated slurry create a unique environment not adequately captured by existing static corrosion models.

Recent advances in mechanochemistry have revealed that mechanical activation can induce catalytic oxidation on iron surfaces [20]. In summary, while a considerable body of research exists on oxidation and corrosion at room temperature, several questions remain unresolved. Most studies suggest that atmospheric corrosion rates are generally low under static conditions. However, recent observations indicate that the reoxidation rate of DRI powder can be significantly accelerated during wet grinding. To gain a deeper understanding of the underlying mechanisms, a comprehensive analysis of the oxidation behavior under dynamic milling conditions is warranted.

## 2. Materials and Methods

### 2.1. Materials

The DRI powder feed was obtained by crushing DRI mini-pellets to 100%, passing a 1 mm sieve. Its chemical composition is given in Table 1.

Wet grinding was performed using an XMQ 240 × 90 conical ball mill (Changsha Tianchuang Powder Technology Co., Ltd., Changsha, China) at a rotation speed of 96 rpm. The grinding media consisted of stainless steel balls with a size distribution of 30 wt.% ⌀20 mm, 40 wt.% ⌀15 mm, and 30 wt.% ⌀10 mm. The ball-to-powder mass ratio was 2:1, and the slurry was prepared at a mass concentration of 50 wt.% using tap water at room temperature (25 ± 2 °C). The slurry pH was monitored but not controlled, remaining at approximately 7.2. The mill was operated under an ambient air atmosphere. After grinding for a designated time (20, 25, 30, or 40 min), the slurry was immediately subjected to magnetic separation using a high-gradient magnetic separator at an optimal intensity of 2.0 kA/m. The beneficiated iron powder was then promptly dewatered using a vacuum filter and dried in a vacuum oven at 60 ± 2 °C for 1.5 h to minimize post-grinding oxidation. The metallization ratio (MR) of the beneficiated product was calculated according to Equation (1):

(1) $$MR=M_{Fe}/T_{Fe}\times 100\%$$

where $T_{Fe}$ is the total iron content in the iron powder (wt.%), and $M_{Fe}$ is the metallic iron content (wt.%). The measurements of $M_{Fe}$ and $T_{Fe}$ were performed using the FeCl_3_-K_2_Cr_2_O_7_ and TiCl_3_-K_2_Cr_2_O_7_ volumetric methods [21], respectively.

The Grade and Recovery of the magnetic separation product were determined by Equations (2) and (3):

(2) $$Grade=M_{1}∕M_{0}\times 100\%$$

(3) $$Recovery=M_{1}∕M_{2}\times 100\%$$

where $M_{0}$ is the total mass of the beneficiated iron powder product (g), $M_{1}$ is the mass of iron in the beneficiated product (g), and $M_{2}$ is the mass of iron in the initial DRI powder feed (g).

The relative oxidation degree (ROD) of the iron powder during grinding was defined by Equation (4):

ROD = (M_DRI_ − M_Fe_)/M_DRI_ × 100% (4)

where M_DRI_ was the metallic iron content in the direct reduced iron powder before grinding.

It should be noted that certain slurry-chemistry variables remained uncontrolled in this study, including dissolved oxygen concentration, the detailed ionic composition of the tap water, and potential galvanic effects arising from stainless steel media wear. These factors may influence the oxidation kinetics and should be systematically investigated in future work to fully decouple the contributions of milling and post-processing.

### 2.2. Methods

The phase composition of samples was characterized by X-ray diffraction (XRD) using a D/max 2550 PC diffractometer (Rigaku Corporation, Tokyo, Japan) with Cu Kα radiation (λ = 1.5406 Å) over a 2θ range of 5° to 80°.

X-ray photoelectron spectroscopy (XPS) was conducted using an ESCALAB 250 X-ray spectrometer (Thermo Scientific, Waltham, MA, USA) with a monochromatic Al Kα source (hv = 1486.6 eV). The base pressure in the analysis chamber was below 1 × 10^−9^ mbar. Survey spectra were recorded with a pass energy of 100 eV, and high-resolution spectra for Fe 2p, Fe 3p, Fe 3s, O 1s, and C 1s were recorded with a pass energy of 20 eV. All binding energies were calibrated by referencing the C 1s peak of adventitious carbon to 284.8 eV. This ex situ analysis was performed on samples after they had been exposed to ambient conditions during handling and transfer. Peak fitting was performed using CasaXPS software with a Shirley background subtraction. Peaks were fitted using a mixed Gaussian-Lorentzian line shape, and the full width at half maximum (FWHM) for each component in a given core level spectrum was constrained to be within ±0.2 eV.

The microstructure and elemental distribution of the iron powder were investigated using a field-emission scanning electron microscope (FE-SEM, Quanta 250 FEG, FEI, Hillsboro, OR, USA) equipped with an energy-dispersive X-ray spectroscopy (EDS) detector (EDAX Inc., Mahwah, NJ, USA). For each sample, a minimum of 10 randomly selected fields of view were examined, and representative images and EDS maps are presented. Particle size distribution was determined by laser diffraction particle size analysis (Mastersizer 3000, Malvern Panalytical, Worcestershire, UK).

The Fe/O atomic ratio was calculated from EDS point analyses by quantifying the atomic concentrations of Fe and O, excluding regions dominated by silica (where Si and O were co-localized without Fe). For each sample, at least 10 particles were analyzed to obtain statistically representative values.

## 3. Results and Discussion

### 3.1. Chemical Composition and Characterization of Feed Materials

Table 1 presents the chemical composition of the DRI powder. The material is characterized by a high silica content (49.76 wt.%) and a relatively low total iron grade (34.95 wt.%). The metallic iron (Fe^0^) content is 29.65 wt.%, resulting in a metallization ratio (MR) of 84.83%. The sum of all components is approximately 100%, with minor elements accounting for the balance. Loss on ignition (LOI) represents the mass loss upon heating at 950 °C, primarily due to the combustion of carbon and volatilization.

**Figure 1:**
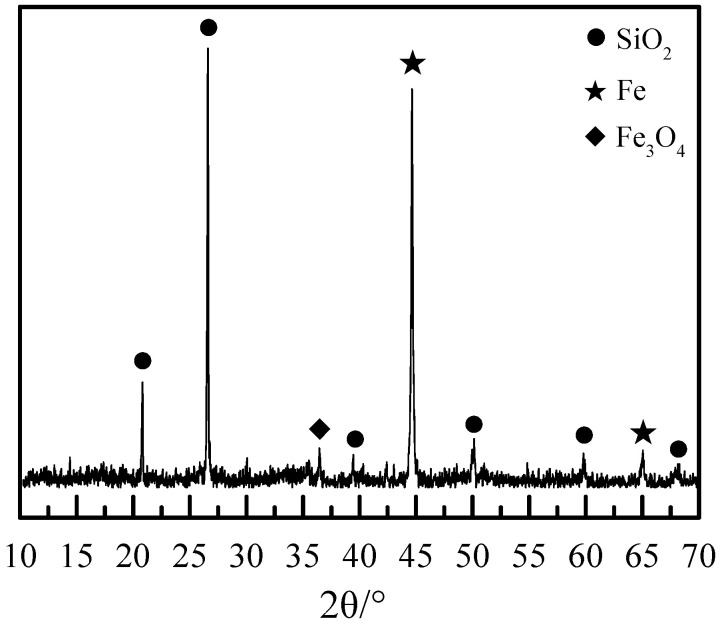
XRD pattern of the DRI powder.

Figure 1 shows the XRD pattern of the DRI powder, confirming that the primary phases are metallic iron (Fe^0^) and quartz (SiO_2_). A very weak peak corresponding to magnetite (Fe_3_O_4_) is also discernible, indicating a minor degree of oxidation or incomplete reduction in the feed material, which is below the detection limit for reliable quantification by chemical analysis in Table 1.

**Figure 2:**
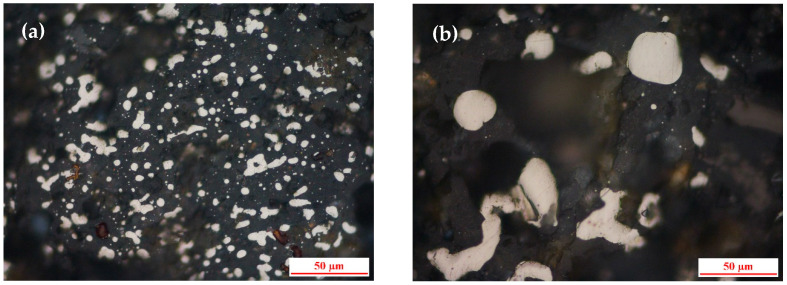

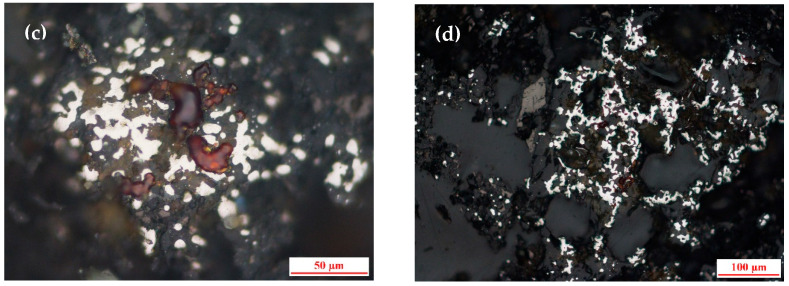
Optical microscopy images of the DRI powder before grinding: (**a**,**b**) fine iron particles embedded in gangue; (**c**,**d**) irregularly shaped iron aggregates.

Figure 2 shows the morphology of metallic iron in the unground DRI powder. The iron particles are mostly small (10–50 μm) and are intimately embedded within the gangue (silicate) phase, as seen in Figure 2a,b. Some larger, irregularly shaped aggregates are also present (Figure 2c,d), which are expected to fracture into finer particles during the ball-milling process.

### 3.2. Determination of Phase Composition of Iron Powder

#### 3.2.1. Grinding and Separating Index

**Figure 3:**
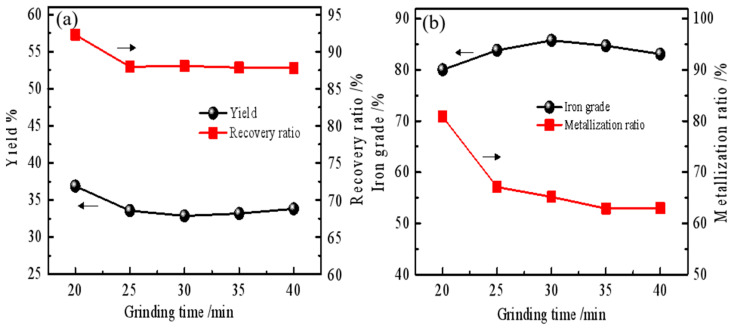
Effect of grinding time on magnetic separation indexes. (**a**) presents the yield and recovery rate as a function of grinding time, while (**b**) shows the iron powder grade and metallization rate as a function of grinding time.

Figure 3 presents the magnetic separation indices as a function of grinding time. Both the product yield and iron recovery decrease with increasing grinding time (Figure 3a). The metallization ratio (MR) of the iron powder shows a marked decline, from 80.83% after 20 min to 62.99% after 40 min (Figure 3b), while the iron grade improves slightly by 3.04%. This trend suggests a loss of metallic iron content as grinding progresses.

**Figure 4:**
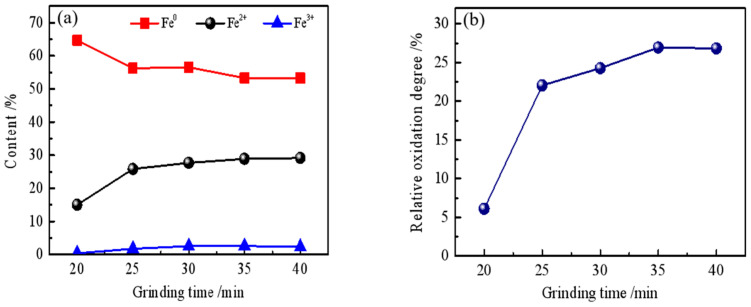
Effect of grinding time on the quality of the iron powder: (**a**) iron phase composition; (**b**) relative oxidation degree (ROD).

To accurately quantify the phase transformation, chemical phase dissolution analysis was performed, with the results shown in Figure 4a. The data in Figure 4a represent the mass percentages of iron in different valence states (Fe^0^, Fe^2+^, and Fe^3+^) relative to the total sample mass, which includes gangue minerals such as silica. Therefore, the sum of Fe^0^, Fe^2+^, and Fe^3+^ at any given grinding time equals the total iron content (TFe) of the sample (approximately 35 wt.%, as shown in Table 1). As the grinding time increases from 20 to 40 min, the fraction of Fe^0^ decreases, accompanied by an increase in Fe^2+^ (from FeO and Fe_3_O_4_) and a slight increase in Fe^3+^ (from Fe_3_O_4_ and Fe_2_O_3_). The calculated relative oxidation degree (ROD) of the iron powder correspondingly increases sharply from 6.08% to 26.81% (Figure 4b). This confirms that oxidation is indeed taking place during the overall process.

#### 3.2.2. SEM-EDS Analysis

**Figure 5:**
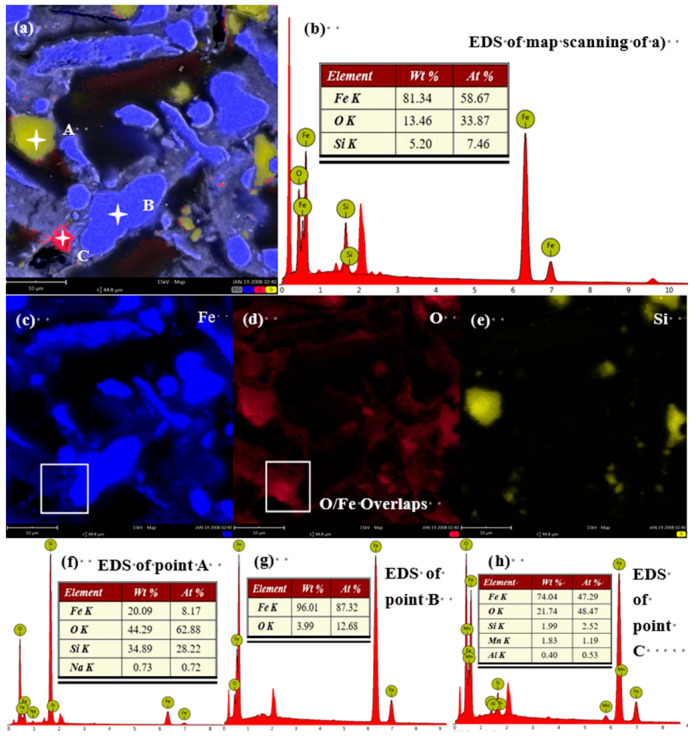
SEM-EDS results for iron powder ground for 20 min: (**a**,**b**) SEM images; (**c**) Fe map; (**d**) O map; (**e**) Si map; (**f**–**h**) EDS point analyses (Blue: Fe; Red: O; Yellow: Si).

**Figure 6:**
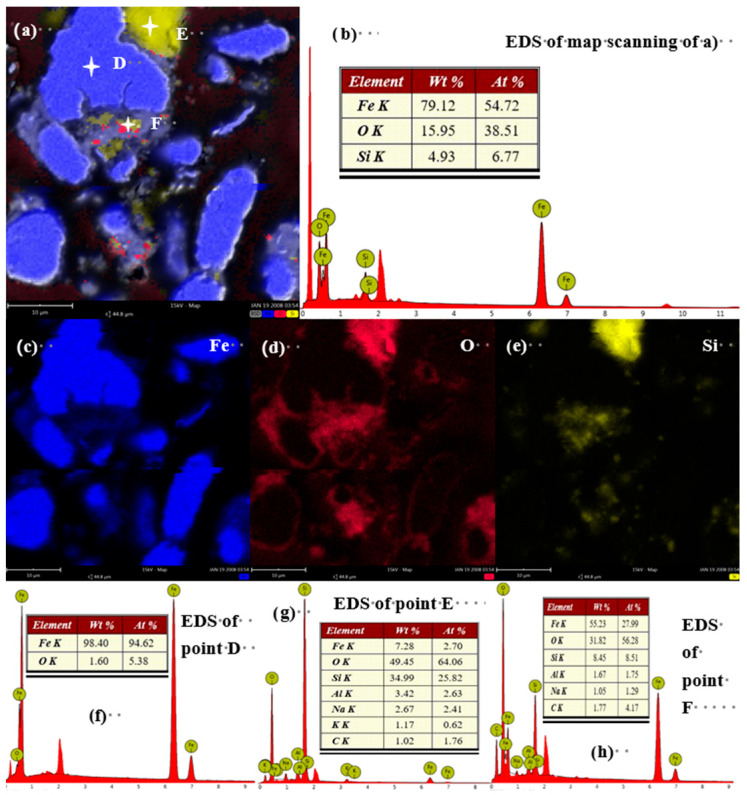
SEM-EDS results for iron powder ground for 30 min. (**a**) SEM image of iron powder after 30 min of milling; (**b**) EDS mapping results of the iron powder after 30 min of milling; (**c**) Fe element mapping of (**a**,**d**) O element mapping of (**a**,**e**) Si element mapping of (**a**,**f**–**h**) point analysis results of spots D, E, and F in Figure 6a, respectively.

**Figure 7:**
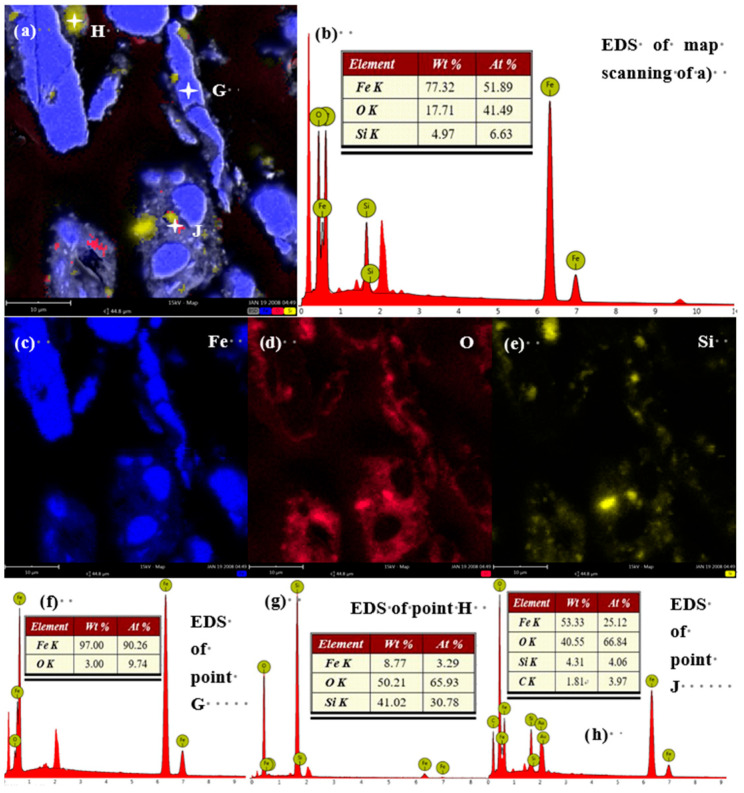
SEM-EDS results for iron powder ground for 40 min. (**a**) SEM image of iron powder after 40 min of milling; (**b**) EDS mapping results of the iron powder after 40 min of milling; (**c**) Fe element mapping of (**a**,**d**) O element mapping of (**a**,**e**) Si element mapping of (**a**,**f**–**h**) point analysis results of spots H, G, and J in Figure 7a, respectively.

Figure 5, Figure 6 and Figure 7 show representative SEM images and corresponding EDS elemental maps for iron powder samples ground for 20, 30, and 40 min. A consistent observation across all samples is that the degree of oxidation is strongly correlated with particle size. In the sample ground for 20 min (Figure 5), smaller iron particles (<10 μm) show a higher degree of oxygen co-localization (Figure 5d) compared to larger particles (10–30 μm). EDS point analysis (Figure 5g,h) confirms significantly higher oxygen content in these fine particles.

**Figure 8:**
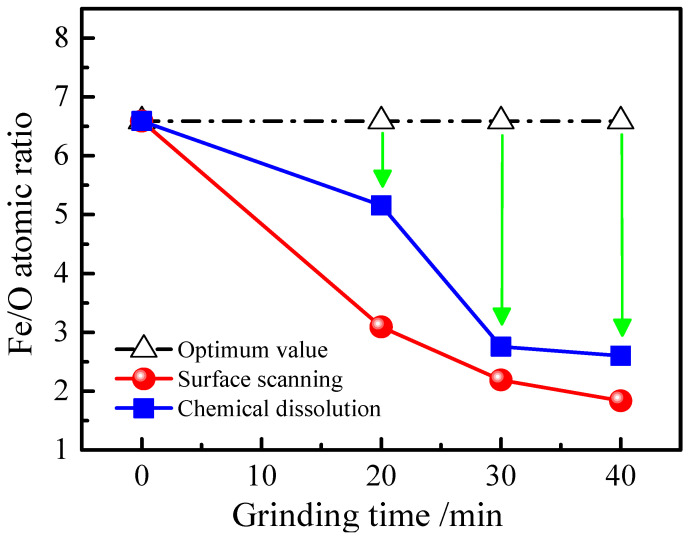
Fe/O atomic ratios of iron powder ground for different milling times.

This trend intensifies with grinding time. After 30 min (Figure 6) and more prominently after 40 min (Figure 7), the oxygen signal is not only associated with fine particles but also begins to appear at the edges of larger iron particles, suggesting progressive oxidation from the surface inward. The Fe/O atomic ratio, calculated from EDS point analyses excluding areas of pure silica, decreases from 6.58 in the feed to 1.83 after 40 min of grinding (Figure 8). These EDS mapping results, derived from multiple examined fields, are consistent with the bulk chemical dissolution data (Figure 4), suggesting that the observed local oxidation patterns are indicative of the overall oxidation trend in the samples.

#### 3.2.3. XRD Analysis

**Figure 9:**
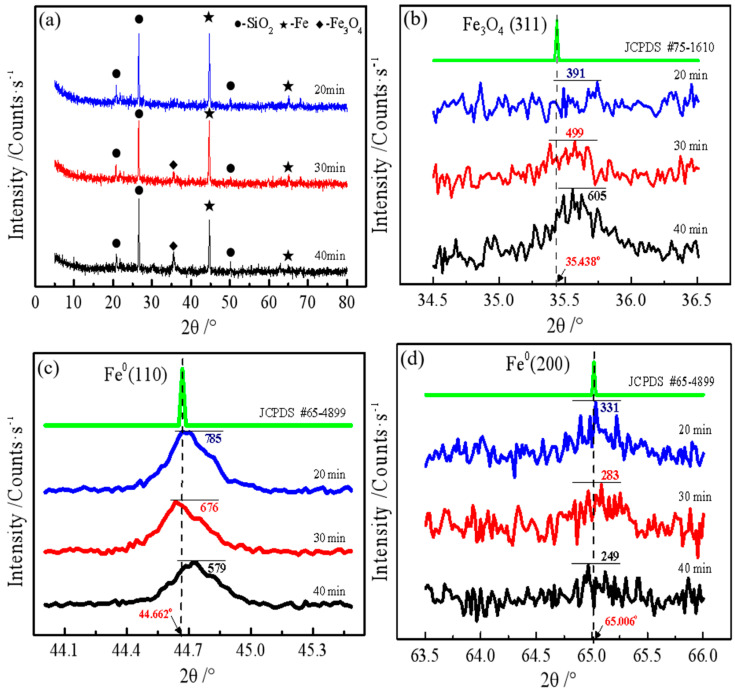
XRD patterns of iron powder obtained at different milling times.

As illustrated in Figure 9, we conducted an X-ray diffraction (XRD) analysis of iron powder (a beneficiation product) subjected to varying milling durations to elucidate the iron phase transformation rules associated with milling time. As depicted in Figure 9a, the primary iron phase is elemental Fe, prominently characterized by the dominance of the Fe (110) crystal plane, supported by the PDF standard card No. 65-4899, with the Fe (100) plane (Fe (200) PDF #65-4899, 65.006°) also present. Notably, a peak is evident in the diffraction spectrum of the 40-min sample at approximately 35.5°, which corresponds to the Fe_3_O_4_ phase (Fe_3_O_4_ (311) PDF #75-1610, 35.438°) [22]. As the milling duration extends, the intensities of the diffraction peaks associated with the Fe (110) and Fe (200) crystal planes exhibit a consistent decline, whereas the intensity of the diffraction peak for the Fe_3_O_4_ (311) crystal plane progressively rises. This observation aligns with the chemical dissolution findings presented in Figure 4, underscoring the dynamic transformation of the material’s crystalline structure under prolonged mechanical milling.

Furthermore, Figure 9b demonstrates that the Fe_3_O_4_ peak intensities significantly increased from 391 counts per second (cps) to 605 cps as the milling time extended from 20 to 40 min. Additionally, Figure 9c illustrates that the peak intensities of Fe (110) in the iron powder milled for 20, 30, and 40 min were 785 cps, 676 cps, and 579 cps, respectively. Moreover, Figure 9d shows that the intensities of the Fe (200) diffraction peaks progressively diminished with increasing milling time, despite their relatively low initial intensity.

Collectively, these results indicate the transformation of a portion of metallic iron (Fe^0^) into magnetite (Fe_3_O_4_) during the milling process, setting the stage for a deeper exploration of the underlying reaction mechanisms in the subsequent section.

#### 3.2.4. XPS Analysis

**Figure 10:**
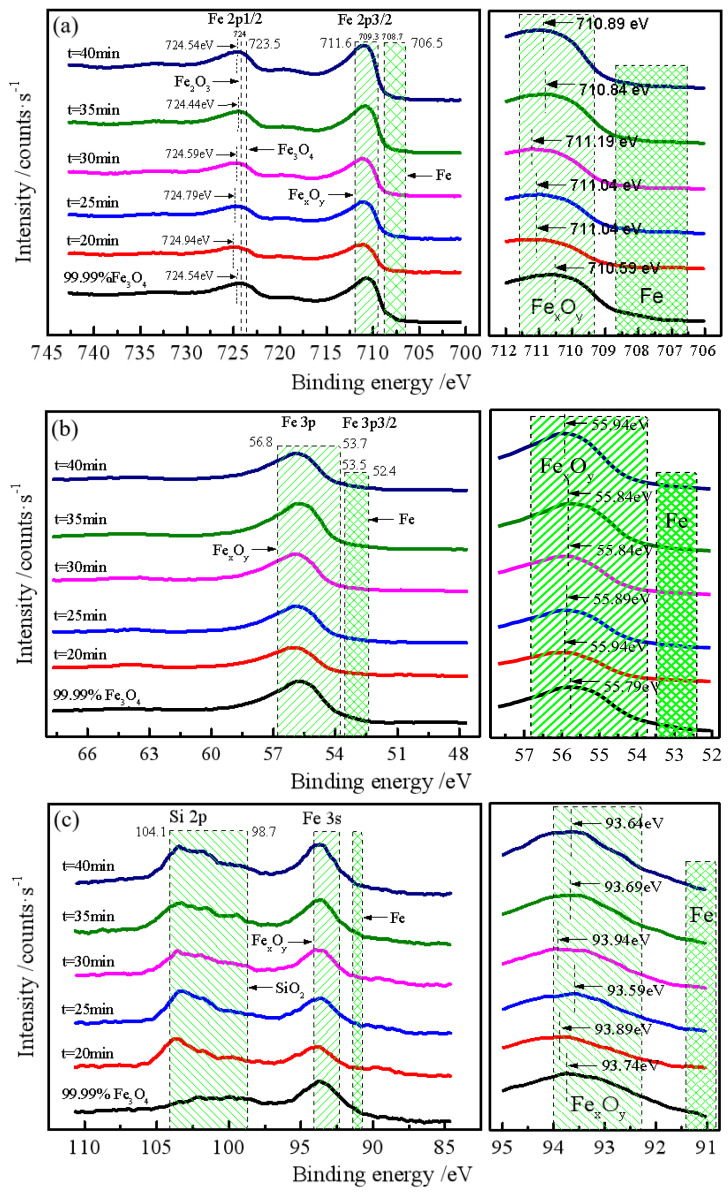

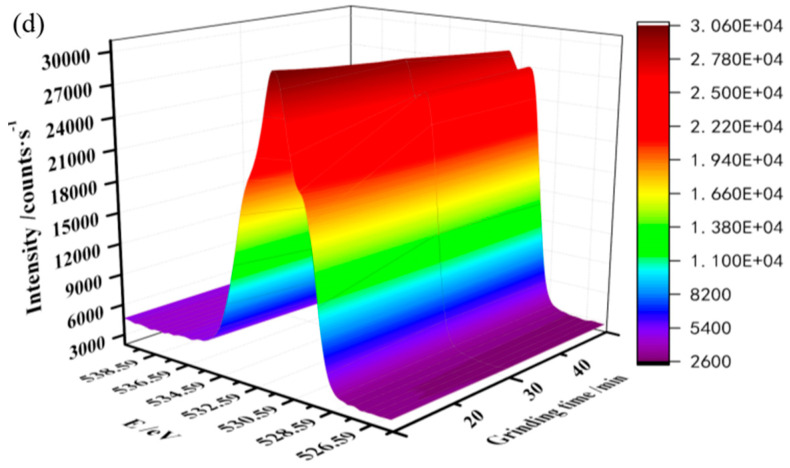
XPS spectra of iron powder under different grinding times: (**a**) Fe 2p; (**b**) Fe 3p; (**c**) Fe 3s; (**d**) O 1s.

The surface chemistry of the iron powder was investigated using XPS. Figure 10 presents the XPS spectra for Fe 2p, Fe 3p, Fe 3s, and O 1s. Due to the well-known complexities in XPS quantification—including spectral overlap and the lack of appropriate standard materials for all iron oxide and hydroxide phases—this analysis focuses on qualitative phase identification. The primary objective is to compare the surface species with the bulk phases identified by chemical dissolution and XRD.

In the Fe 2p spectrum (Figure 10a), a peak appears in the Fe 2p_1_/_2_ region at binding energies ranging from 724.44 to 724.94 eV across different grinding times. These values are closer to the characteristic peak of Fe_2_O_3_ (724.00 eV) than to that of Fe_3_O_4_ (723.50 eV), suggesting the presence of ferric oxide species on the surface. The Fe 2p_3_/_2_ region exhibits peaks between 710.89 and 711.19 eV, which are higher than the standard value for Fe_3_O_4_, further supporting the formation of Fe^3+^-rich phases.

The Fe 3p spectra (Figure 10b) show peaks in the energy range of 55.84–55.94 eV, characteristic of Fe_2_O_3_. In the Fe 3s spectra (Figure 10c), peaks at 93.59–93.94 eV suggest the presence of Fe_3_O_4_ and FeO. The O 1s spectra (Figure 10d) reveal multiple oxygen species: a peak at 529.1–530.7 eV corresponds to lattice oxygen (O^2−^) in iron oxides, while a broader feature at 531.0–532.8 eV indicates the presence of hydroxyl groups (OH^−^) from iron hydroxides such as Fe(OH)_3_ and FeOOH. An additional contribution at 532.1–534.3 eV is attributed to oxygen in residual silica (SiO_2_) that co-migrated with iron particles during magnetic separation.

These XPS observations provide qualitative evidence that the nanoscale surface of the iron powder consists of a complex mixture of Fe_2_O_3_, Fe_3_O_4_, Fe(OH)_3_, and FeOOH—a composition distinctly different from the bulk Fe/Fe_3_O_4_ phases identified by chemical dissolution and XRD. The consistent trend of surface oxidation across different core levels and grinding times supports the conclusion that an oxidized/hydroxylated surface layer forms during the wet grinding process.

### 3.3. Analysis of Oxidation Mechanisms

**Figure 11:**
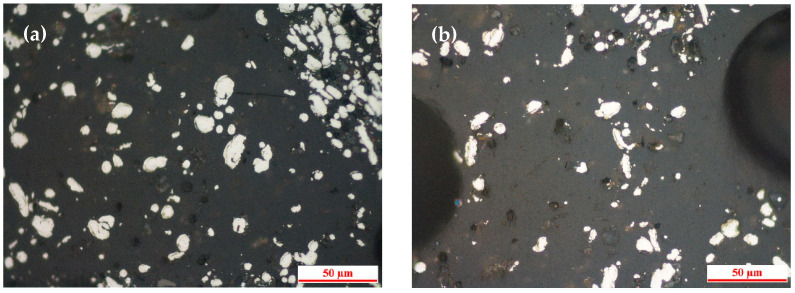

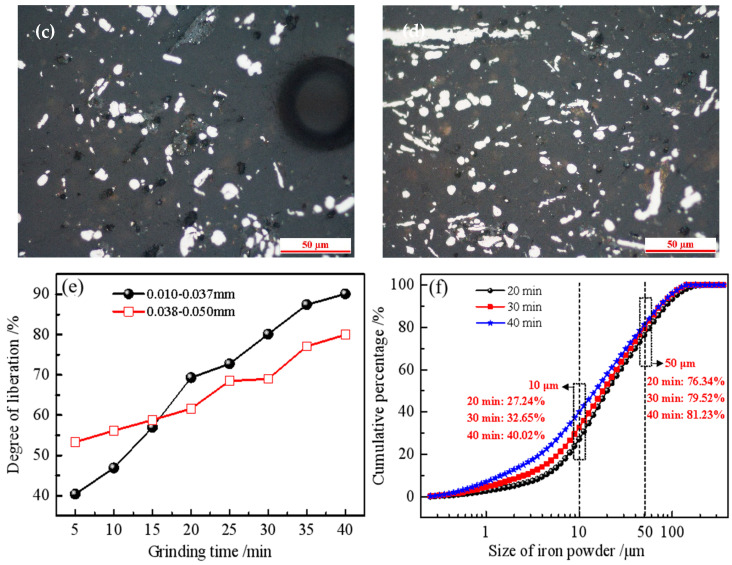
Effect of grinding time on particle size of iron powder: (**a**) 20 min; (**b**) 25 min; (**c**) 30 min; (**d**) 40 min; (**e**) liberation degree; (**f**) size distribution.

Figure 11 illustrates the morphological features, size distribution, and liberation degree of the iron powder obtained. As shown in Figure 11a, metallic iron particles exhibit droplet-like, spherical, and punctate shapes, similar to those in the reduced pellets prior to grinding. Figure 11b–d reveal that as grinding time increases, the iron particles progressively diminish in size and become increasingly irregular in shape, indicating continuous compression and deformation. Notably, Figure 11f demonstrates a marked increase in the cumulative percentage of <10 μm particles, rising from 27.24% to 40.02%, while the cumulative percentage of <50 μm particles marginally increases from 76.34% to 81.23% within the 20–40 min grinding interval. This suggests that extended grinding time significantly enhances the production of fine iron particles, thereby exacerbating reoxidation.

Furthermore, process mineralogy analysis (Figure 11e) reveals that the liberation degree of 10–37 μm particles improves from 40.36% to 90.13% over the 5–40 min grinding period. Similarly, the liberation degree of 37–50 μm particles increases from 53.34% to 79.95% during the same interval. It is particularly noteworthy that the reoxidation phenomenon intensifies at 25 min of grinding (Figure 4b), when the liberation degrees of 10–37 μm and 37–50 μm particles reach 72.74% and 68.49%, respectively. This indicates that reoxidation significantly worsens once the metallic iron surface is nearly entirely exposed.

Based on the principles of nucleation kinetics and the thermodynamics of metallic iron grain growth, the reduction of iron oxides and subsequent grain growth involve three critical stages: the chemical reaction stage, the nucleation stage, and the crystal growth stage [23,24]. During the crystal growth phase, reductions in temperature and time are paramount. Consequently, the size of metallic iron particles produced through a rapid, low-temperature reduction process (10–50 μm) is significantly smaller than those obtained via conventional pyrometallurgical coal-based direct reduction (75–200 μm). This disparity in particle size can lead to substantial differences in the physicochemical properties of the iron powders.

Table 2 provides a detailed comparison of the physical properties of 100 g of spherical iron powder particles with varying diameters. As illustrated, the energy-efficient process yields 400 to 8000 times more iron powder particles than the traditional method. Additionally, the total surface area ratio between the two processes ranges from 3000 to 160,000, which may explain the rapid oxidation observed in these materials.

The wet grinding process facilitates the gradual separation of iron particles from gangue minerals, with the continuous impact and grinding action of stainless steel balls continually exposing fresh surfaces to the solid–liquid–gas interface. Mechanical activation also affects the reducibility and oxidation behavior of iron oxides [25]. Research has demonstrated that the grinding process significantly influences the electrochemical properties of mineral surfaces [15]. This process generates electrical contact between diverse mineral particles, leading to electrochemical reactions where regions of high electrostatic potential act as cathodes (promoting reduction reactions), while regions of low electrostatic potential serve as anodes (initiating oxidation reactions).

Furthermore, metallic iron reacts with dissolved oxygen and other chemical constituents in the slurry, resulting in atmospheric corrosion. Both atmospheric and electrochemical corrosion processes are intricately linked to the roles played by O_2_ and H_2_O in the wet grinding environment. Following oxidation, metallic iron undergoes immediate hydrolysis and precipitation, forming an iron oxide layer on the surface of the iron particles.

**Figure 12:**
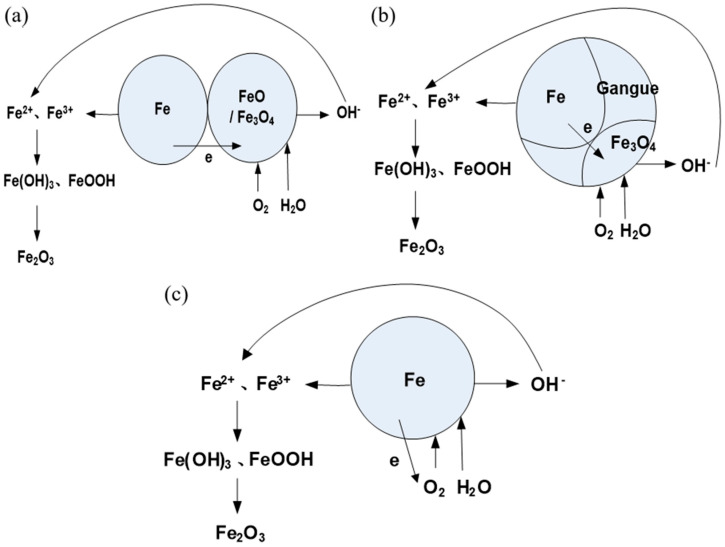
Schematic diagram of the interface reactions of DRI powder during the grinding process: (**a**,**b**) electrochemical corrosion; (**c**) atmospheric corrosion.

Figure 12 illustrates a schematic representation of the interface reactions occurring in DRI powder during the grinding process. The oxidation mechanisms in the aqueous slurry can be described by standard electrochemical reactions:

(a) Electrochemical corrosion (galvanic coupling)

Anodic reaction (oxidation):

Fe = Fe^2+^ + 2e^−^ (5)

Cathodic reaction (reduction): In an aerated, near-neutral pH slurry, the primary cathodic reaction is oxygen reduction:

O_2_ + 2H_2_O + 4e^−^ = 4OH^−^ (6)

Overall reaction: The ferrous ions (Fe^2+^) produced anodically react with hydroxide ions (OH^−^) to form ferrous hydroxide:

2Fe + O_2_ + 2H_2_O = 2Fe(OH)_2_ (7)

(b) Atmospheric Corrosion and Further Oxidation

Ferrous hydroxide is unstable and further oxidizes in the presence of oxygen:

4Fe(OH)_2_ + O_2_ = 2Fe_2_O_3_ + 4H_2_O (8)

The ferric ions (Fe^3+^) can also hydrolyze and precipitate as various oxyhydroxides, depending on local pH and ion concentration.

Fe^3+^ + 3OH^-^ = Fe(OH)_3_ (9)

Fe(OH)_3_ = FeOOH + H_2_O (10)

The Fe_3_O_4_ detected by XRD in the bulk is likely formed via the reaction between Fe^2+^ and Fe^3+^ species (the Schikorr reaction [26,27]) or by the solid-state transformation of Fe(OH)_2_.

Fe^2+^ + 2Fe^3+^ + 8OH^−^ = Fe_3_O_4_ + 4H_2_O (11)

The oxidation rate is likely controlled by a combination of factors. In the initial stages, when fresh surfaces are constantly exposed, the reaction may be activation-controlled, limited by the charge transfer rate at the interface. As an oxide/hydroxide layer builds up, the process may become diffusion-controlled, limited by the transport of oxygen or ions through the growing surface layer. Kinetic transitions of this type have been modeled in related reduction systems using the random pore model [28]. The near-neutral pH (7.2) of the slurry favors the formation of hydroxide and oxyhydroxide species, consistent with our XPS findings.

## 4. Conclusions

This study systematically investigated the impact of wet ball-milling grinding time on the oxidation behavior of DRI powder. The following main conclusions are drawn:

(1) Oxidation of direct reduced iron powder occurs during the wet grinding and subsequent handling stages. While the experimental procedure was designed to minimize post-grinding oxidation, we acknowledge that some oxidation may occur during sample handling and drying. The observed increase in ROD with grinding time (from 6.08% to 26.81%) reflects the combined effects of mechanical activation during milling and exposure during post-processing. SEM-EDS analysis reveals that the oxidation phenomenon is more extensive in smaller particles (<10 μm). XRD analysis demonstrates that part of the Fe^0^ gradually transforms to Fe_3_O_4_ during grinding, a finding that aligns with the chemical analysis results.
(2) XPS analysis of the Fe 2p, Fe 3p, Fe 3s, and O 1s core levels provides qualitative evidence that Fe_2_O_3_, Fe_3_O_4_, Fe(OH)_3_, and FeOOH are the main iron phases on the nanoscale surface of the iron powder. This surface composition is distinctly different from the bulk Fe/Fe_3_O_4_ phases identified via chemical dissolution, SEM-EDS, and XRD analysis.
(3) The reaction between zero-valent iron and dissolved oxygen, along with other factors including iron surface defects, mechanical activation, and the mode of grinding, may all affect the oxidizability of the iron powders. The slurry environment (including dissolved oxygen and pH) plays a critical role in determining the oxidation kinetics, although these parameters were not fully controlled in the present study. Recent advances in electrically driven hydrogen reduction, such as the fully electrically driven process reported by Liang et al. [29] for zero-carbon DRI production, may provide alternative pathways to produce DRI with enhanced oxidation resistance. Future work will examine these factors in detail, aiming to develop effective measures for inhibiting oxidation during processing.

## Figures and Tables

**Table 1:** Chemical composition of the DRI powder (mass, %).

TFe	Fe	SiO_2_	FeO	Al_2_O_3_	C
34.95	29.65	49.76	6.82	5.84	2.81
CaO	MgO	S	P	Cl	LOI
0.71	0.44	0.03	0.09	0.05	2.41

**Table 2:** Comparison of physical properties of 100 g of spherical iron powder particles with different diameters. Values in parentheses represent the ratio relative to the 200 μm particle.

Diameter (μm)	Iron Powder Number	Specific Surface Area of Single Particle (cm^2^·g^−1^)	Total Surface Area (Arbitrary Units)
10	24,267,590 (8000)	76.24	18,501,339 (160,000)
26.34 (Avg. at 20 min)	1,327,943 (437.8)	28.94	384,362 (3324.0)
75	59,887 (19.7)	10.30	6170 (53.4)
150	7190 (2.4)	5.08	365 (3.2)
200	3033 (1.0)	3.81	116 (1.0)

## Data Availability

The original contributions presented in this study are included in the article. Further inquiries can be directed to the corresponding author.

## References

[1] Srivastava U., Kawatra S.K. (2009). Strategies for processing low-grade iron ore minerals. Miner. Process. Extr. Metall. Rev..

[2] Song S.X., Campos-Toro E.F., Zhang Y.M., Lopez-Valdivieso A. (2013). Morphological and mineralogical characterizations of oolitic iron ore in the Exi region, China. Int. J. Miner. Metall. Mater..

[3] Wang Y., Xing S.W., Zhang Z.J., Ma Y.B., Zhang Y. (2014). Reserves analysis of identified low-grade iron resources in China. Multipurp. Util. Miner. Resour..

[4] Yu Y.F. (2005). Processing state and technology progress of iron ore in China. Conserv. Util. Miner. Resour..

[5] Sun Y.S., Han Y.X., Li Y.F., Li Y.J. (2017). Formation and characterization of metallic iron grains in coal-based reduction of oolitic iron ore. Int. J. Miner. Metall. Mater..

[6] Huang Z.C., Zhong R.H., Yi L.Y., Jiang T., Wen L.M., Liang Z.K. (2017). Reduction enhancement mechanisms of a low-grade iron ore-coal composite by NaCl. Metall. Mater. Trans. B.

[7] Liang Z.K., Huang Z.C., Yi L.Y., Zhong R.H. (2018). Study on direct reduction of low-grade iron ore-coal mini-pellets in coal-based rotary kiln. Proceedings of the TMS Annual Meeting & Exhibition.

[8] Zuo Y., Wang Z., Li K., Zhang J., Conejo A.N. (2023). Hydrogen-Based Direct Reduction of Iron Oxides: A Review on the Influence of Impurities. Sustainability.

[9] Kieush L., Lesiak S., Rieger J., Leitner M., Schmidt L., Daghagheleh O. (2024). Reoxidation Behavior of the Direct Reduced Iron and Hot Briquetted Iron during Handling and Their Integration into Electric Arc Furnace Steelmaking: A Review. Metals.

[10] De Smit E., van Schooneveld M.M., Cinquini F., Bluhm H., Sautet P., de Groot F.M., Weckhuysen B.M. (2011). On the surface chemistry of iron oxides in reactive gas atmospheres. Angew. Chem. Int. Ed..

[11] Zhang K., Li L.F., Shaikhutdinov S., Freund H.J. (2018). Carbon monoxide oxidation on metal-supported monolayer oxide films: Establishing which interface is active. Angew. Chem. Int. Ed..

[12] El-Sayed M.S.A. (2014). A comparative study on the electrochemical corrosion behavior of iron and X-65 steel in 4.0 wt% sodium chloride solution after different exposure intervals. Molecules.

[13] Martin M., Fromm E. (1997). Low-temperature oxidation of metal surfaces. J. Alloys Compd..

[14] Koryta J., Dvořák J., Kavan L. (1993). Principles of Electrochemistry.

[15] Perez N. (2004). Electrochemistry and Corrosion Science.

[16] Okoye C.O., Zhang Z., Lin J., Liu Y., Li X., Wang S., Matthews D., Evans T.J., Song S., Zhang D. (2024). An Experimental Study of Room Temperature Reoxidation Behavior of Direct H_2_-Reduced Iron in Air. Ind. Eng. Chem. Res..

[17] Gabe D.R. (1978). Principles of Metal Surface Treatment and Protection.

[18] Hauffe K. (1965). Oxidation of Metals.

[19] Landolt D. (2007). Corrosion and Surface Chemistry of Metals.

[20] Jiang C., Wu Y., Zhang Y., Zong J., Wang N., Liu G., Liu R., Yu H. (2025). Supramolecular Modulation for Selective Mechanochemical Iron-Catalyzed Olefin Oxidation. Angew. Chem. Int. Ed..

[21] Saikkonen R.J., Rautiainen I.A. (1993). Determination of ferrous iron in rock and mineral samples by three volumetric methods. Bull. Geol. Soc. Finl..

[22] Su Z.J., Zhang Y.B., Han B.L., Chen Y.M., Lu M.M., Peng Z.W., Li G.H., Jiang T. (2017). Interface reaction between Fe_3-x_Sn_x_O_4_ and CaO roasted under CO-CO_2_ atmosphere. Appl. Surf. Sci..

[23] Mao W.M., Zhao X.B. (1994). Metal Recrystallization and Grain Growth.

[24] Zhu D.Q., Chun T.J., Pan J. (2011). Mechanism of action of improving reduction on low grade hematite pellets by adding nucleating agent. J. Univ. Sci. Technol. Beijing.

[25] Kleonovskii M.V., Sheshukov O.Y., Mikheenkov M.A., Mikheenkov A.M., Matyukhin O.V. (2024). Effect of mechanical processing on reduction of iron oxides in man-made raw materials. Izv. Ferr. Metall..

[26] Yamamoto M., Takamura Y., Kokubo Y., Kato K., Yamada I. (2023). Solid-State Schikorr Reaction from Ferrous Chloride to Magnetite with Hydrogen Evolution as the Kinetic Bottleneck. Inorg. Chem..

[27] Li C., Long Z., Guo D., Wang X., Liu Y. (2023). Catalytic mechanism of the Schikorr reaction promoted by the copper oxide nanosheet during a low-temperature hydrothermal process. Mater. Chem. Phys..

[28] Hosseinzadeh M., Kasiri N., Rezaei M. (2024). Random pore model insights into structural and operational parameters for hydrogen-based iron oxide reduction. Process Saf. Environ. Prot..

[29] Liang Z., Zhang J., Li K., Shao L., Liu Z., Yang Z., Jiang C., Ren S., Conejo A.N. (2025). A fully electrically driven hydrogen direct reduction process for zero-carbon green steel production. Green Chem..

